# An Institutional Approach to the Management of Asymptomatic Chorioamnionitis-Exposed Infants Born ≥35 Weeks Gestation

**DOI:** 10.1097/pq9.0000000000000238

**Published:** 2019-12-05

**Authors:** Arpitha Chiruvolu, Barbara Petrey, Karen C. Stanzo, Yahya Daoud

**Affiliations:** From the * Department of Women and Infants, Baylor Scott & White Medical Center McKinney, Pediatrix Medical Group of Dallas, Tex.; † Department of Women and Infants, Baylor Scott & White Medical Center McKinney, Tex.; ‡ Department of Women and Infants, Baylor Scott & White Medical Center McKinney, Tex.; § Department of Quantitative Sciences, Center for Clinical Effectiveness, Baylor Scott & White Health Care System, Dallas, Tex.

## Abstract

Our newborn practice routinely treated asymptomatic chorioamnionitis-exposed infants born at 35 weeks gestation or greater with empiric antibiotics. Starting April 1, 2017, we implemented an algorithm of not treating, unless there was an abnormal clinical and/or laboratory evaluation. The goal of this quality improvement initiative was to reduce the percentage of chorioamnionitis-exposed infants treated with antibiotics (primary outcome measure) to <50%.

**Methods:**

We compared 123 chorioamnionitis-exposed infants born 1 year before implementation (pre-algorithm group, April 1, 2016, to March 31, 2017) with 111 born 1 year following implementation (post-algorithm group, April 1, 2017, to March 31, 2018). The primary outcome measure was analyzed monthly using a run chart.

**Results:**

The maternal and neonatal characteristics were similar between both groups. Significantly fewer infants in the post-algorithm group received antibiotics compared with the pre-algorithm group (4.5% versus 96.8%; *P* < 0.01). There were no differences in median hospital length of stay or incidence of neonatal intensive care unit admissions between both groups. There were no positive blood cultures or readmissions within 7 days for early-onset sepsis in either group.

**Conclusion:**

An institutional approach of monitoring chorioamnionitis-exposed infants with a clinical and laboratory evaluation decreased antibiotic utilization in the mother-baby unit by 95% without an increase in hospital length of stay, neonatal intensive care unit admissions, or readmissions for early-onset sepsis.

## Abstract 2

Supplemental Digital Content is available in the text.

## INTRODUCTION

Chorioamnionitis is an infection or inflammation of chorion, amnion, or both. Reportedly, it occurs in 3%–10% of all pregnancies.^1,2^ Accurate diagnosis of chorioamnionitis can be challenging, as some of the signs and symptoms overlap with other noninfectious causes, such as epidural anesthesia.^3,4^ Even though the incidence of early-onset sepsis (EOS) in infants exposed to chorioamnionitis is significantly higher than infants not exposed, it is still as low as 1.3 to 7.2 per 1,000 live births.^5–7^ The risk of infection is reduced if mothers receive intrapartum antibiotic prophylaxis.^8,9^ Symptomatic infants born to mothers diagnosed with chorioamnionitis need neonatal intensive care unit (NICU) admission and intravenous (IV) antibiotics. The management of asymptomatic infants born 35 weeks gestation or greater exposed to maternal chorioamnionitis is controversial. In 2010, the Committee on the Fetus and Newborn (COFN) and Centers for Disease Control (CDC) recommended the evaluation of these infants at birth with blood culture and empiric treatment with broad-spectrum antibiotics. Laboratory evaluation included complete blood count (CBC) and/or C-reactive protein (CRP) at birth and/or 6 to 12 hours of life.^10,11^ Antibiotics were to be discontinued after 48 hours if the newborn remains asymptomatic, and the blood culture result is negative. This management strategy may provide a maximum reduction of EOS, but with acceptance of significant overtreatment.^12^ In some institutions, this may involve NICU admission leading to mother-infant separation, increased hospital length of stay (HLOS), and increased healthcare costs.^13^ Today, COFN recommends that institutions should develop approaches that are best suited to their local resources and structures to minimize the duration of antibiotic administration to uninfected infants.^14^

Our newborn practice covering 2 delivery services in the same hospital system followed the CDC 2010 guidelines, and therefore, we treated chorioamnionitis-exposed infants born 35 weeks gestation or greater with empiric antibiotics. To reduce the antibiotic utilization in asymptomatic healthy newborns, we developed and implemented an institutional algorithm, starting April 1, 2017. The objective of this quality improvement (QI) study was to report the effects of that practice change. Our SMART aim statement was by following the new institutional algorithm 1-year post-implementation (April 1, 2017, to March 31, 2018), the percentage of asymptomatic chorioamnionitis-exposed infants treated with antibiotics would decrease to <50% without an increase in NICU admissions or readmissions for EOS.

## METHODS

### Context

This QI project was a collaboration between the 2 delivery services (~3,500 deliveries per year) in a large nonprofit healthcare system. The hospitals have mother-baby units (MBU) staffed by newborn providers. Infants born at 35 weeks gestation or greater room-in with their mothers during the entire hospital stay. Both hospitals have NICUs that directly admit infants born <35 weeks gestation per hospital policy.

### Intervention

Historically, the mother was started on intrapartum antibiotic prophylaxis after the obstetric provider made the clinical diagnosis of chorioamnionitis. During the pre-algorithm period, if the infant born at 35 weeks gestation or greater was asymptomatic at birth, newborn providers obtained a CBC and blood culture and started IV ampicillin and gentamicin therapy. The infant was separated from the mother after birth for a brief period for lab draw and IV placement. If the blood culture was negative and infant appeared clinically well, a total of 6 doses of antibiotics were given over 48 hours (4 doses of ampicillin every 12 hours and 2 doses of gentamicin every 36 hours) while the infant continued to room in with the mother in the MBU. The frequency of routine vital signs measurement was 8 hours. In a few cases, antibiotics were continued beyond 48 hours if the labs (CBC and/or CRP) continued to be abnormal, the blood culture was positive and/or the infant had signs of sepsis. Such infants were transferred to the NICU. This management guideline resulted in ~3% of all newborns receiving empiric antibiotics in the MBU.

**Fig. 1.**
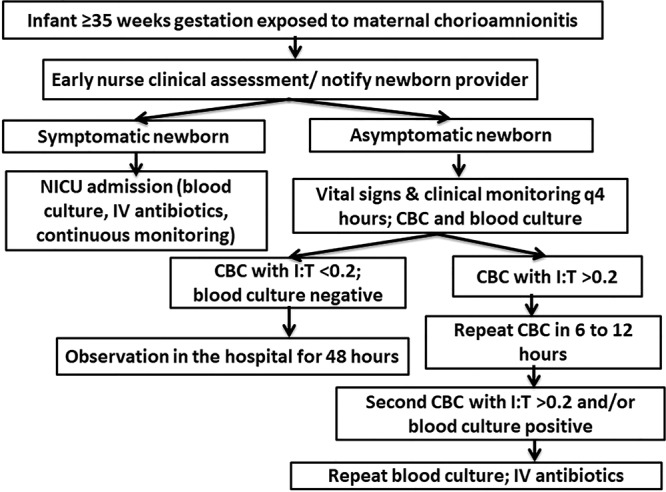
Institutional algorithm for the management of chorioamnionitis-exposed infants.

After a review of the published literature, we developed an institutional algorithm focusing on reducing infant antibiotic utilization in the MBUs without an increase in the healthcare costs, NICU admissions, or readmissions for sepsis. A multidisciplinary group consisting of newborn and obstetric providers, MBU and NICU nursing staff, and neonatal pharmacists were involved in the preparation of the algorithm. Starting April 1, 2017, once the chorioamnionitis-exposed infant was born at 35 weeks gestation or greater, a clinical assessment was performed by the MBU nurse, and the newborn provider was notified. If asymptomatic, a CBC and blood culture were obtained, but the infant was not started on empiric antibiotics. The MBU nurse performed clinical monitoring and vital signs, including temperature, heart rate, and respiratory rate every 4 hours during the entire hospital stay that lasted at least 48 hours. The newborn providers performed comprehensive exams daily until discharge. If initial CBC was abnormal (defined as immature to total neutrophil ratio [I:T] >0.2), it was repeated in 12 hours. If the second I:T was normal and the infant was clinically well, no antibiotics were started. If the second CBC was also abnormal with I:T > 0.2, IV antibiotics were started and administered while the infant continued to room in with the mother in MBU. Antibiotics were discontinued in 48 hours if the blood culture remained negative. If blood culture was positive and/or signs of sepsis were present, the infant was transferred to NICU for continuous monitoring and IV antibiotics. Figure 1 depicts the institutional algorithm.

### Implementation

The obstetric and newborn providers and nursing staff received education via presentations during departmental and staff meetings. We also sent an e-mail communication. Personal educational sessions were provided to the nursing staff, reinforcing the algorithm, clinical assessment of infants, and calculation of I:T. Our interprofessional QI team met monthly to review the outcomes, cases of EOS, and staff feedback.

### Evaluation/Measures

The study group was the total number of infants born to mothers with a clinical diagnosis of chorioamnionitis. The primary outcome measure was the percentage of chorioamnionitis-exposed neonates who received antibiotics, assessed monthly on a run chart. We defined antibiotic exposure as the receipt of at least 1 dose of IV or intramuscular antibiotics within the first 48 hours of life.

The electronic medical record was queried to identify women with the diagnosis of chorioamnionitis. These charts were manually reviewed to confirm the clinical diagnosis made by the obstetric provider. Collected data included maternal demographics and obstetric and fetal complications. Neonatal data included gestational age, birth weight, sex, Apgar scores, and other resuscitation variables. We recorded the laboratory data along with the antibiotic exposure data, including infants receiving antibiotics and antibiotic doses. The newborn outcome data included the need for NICU admission and readmission for EOS within 7 days of discharge. We also collected NICU admission data and HLOS.

Study subjects included inborn infants born at 35 weeks gestation or greater and a maternal clinical diagnosis of chorioamnionitis. Exclusion criteria were major birth defects or birth <35 weeks gestation, both of which require direct admission to the NICU. We defined cases of culture-confirmed EOS as positive blood or cerebrospinal fluid culture of a single known pathogenic species within the first 5 days of incubation. Cultures growing common skin flora (including *Staphylococcal epidermidis*) or multiple organisms were considered contaminants.

### Data Analysis

Data were collected over 2 periods. The pre-algorithm period was 1 year, April 1, 2016, to March 31, 2017. The post-algorithm period was also 1 year, from April 1, 2017, to March 31, 2018. The pre-algorithm period reflects the use of the 2010 CDC guidelines, whereas the post-algorithm period reflects the use of the institutional algorithm.

The primary outcome measure was analyzed monthly using a run chart. We analyzed continuous data as mean and SD if parametrically distributed or median and 25th to 75th interquartile range if non-parametric, and other variables as counts (percent). We compared data between the pre- and post-algorithm groups with the use of the two-tailed Student *t* test for continuous variables parametrically distributed and Wilcoxon/Kruskal-Wallis rank-sum tests for nonparametric distribution. Pearson’s chi-squared/Fisher exact tests were used for categorical variables. A probability value <0.05 was considered to be the threshold of statistical significance. We performed statistical analyses with JMP (version 13.2; SAS Institute Inc., Cary, N.C.). An MS Excel monthly run chart was used to depict the primary outcome measure (percentage of chorioamnionitis-exposed neonates who received antibiotics).

### Ethical Considerations

The local institutional review board reviewed the project and determined to be a local QI project and did not meet the definition of human subjects’ research.

## RESULTS

During the pre-algorithm study period, out of 3,701 deliveries, 35 weeks gestation or greater, 123 (3.3%) were complicated by maternal chorioamnionitis. During the post-algorithm study period, out of 3,240 deliveries, 35 weeks gestation or greater, 111 (3.4%) were complicated by maternal chorioamnionitis. Maternal demographics and characteristics were similar between both groups (Table 1). There were no significant differences in neonatal characteristics between both groups (Table 2). In the pre-algorithm group, 119 of 123 (96.8%) infants received antibiotics, whereas 5 of 111 (4.5%) infants received antibiotics in the post-algorithm group (*P* < 0.01). There was 100% compliance with antibiotic therapy according to the algorithm. The median antibiotic doses decreased from 6 (0–14) in the pre-algorithm group to 0 (0–6) in the post-algorithm group (*P* < 0.01). None of the blood cultures were positive. We observed no differences in the incidence of NICU admissions or HLOS. None of the infants were readmitted for sepsis within 7 days of discharge.

**Table 1.**
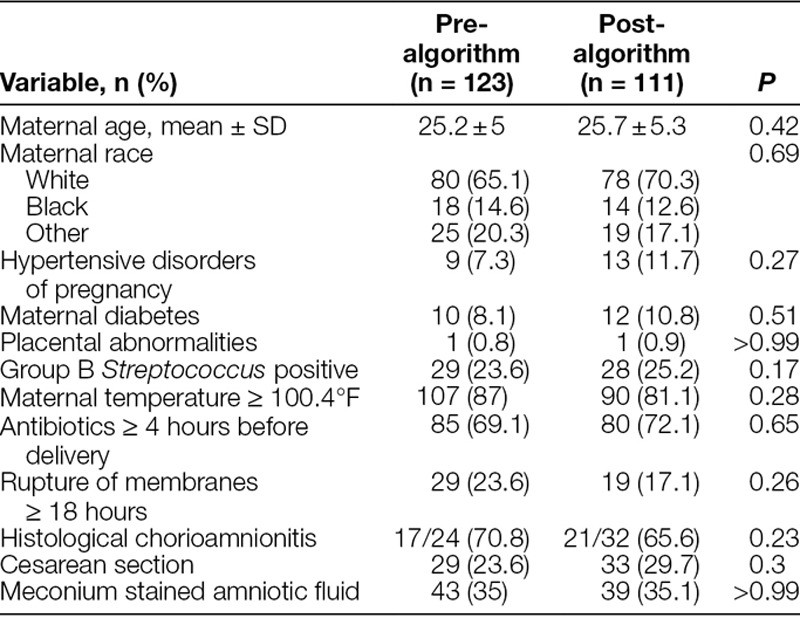
Maternal Characteristics

**Table 2.**
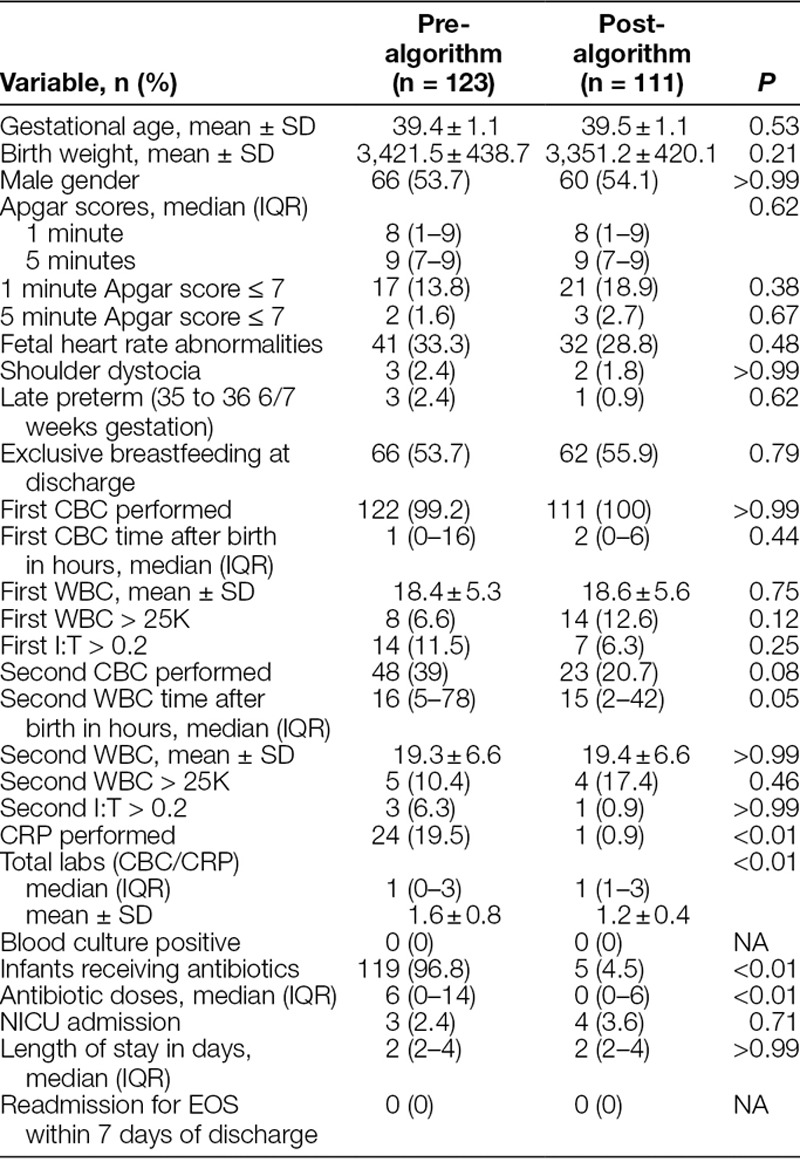
Neonatal Characteristics and Outcomes

**Fig. 2.**
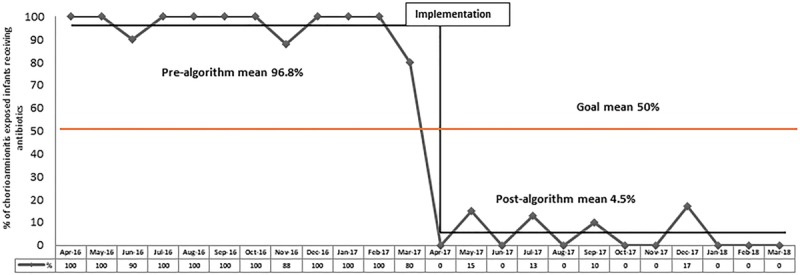
Monthly run chart pre- and post-algorithm implementation. The percentage of chorioamnionitis-exposed infants treated with antibiotics decreased by 95% (96.8% pre-algorithm to 4.5% post-algorithm).

The monthly run chart (Fig. 2) depicts the primary outcome measure of the percentage of chorioamnionitis-exposed neonates who received antibiotics. A Statistical Process Control chart is also presented (see Supplemental Digital Content at http://links.lww.com/PQ9/A148 for Figure 1).

There were 3 infants with clinical signs of sepsis in the pre-algorithm group and 4 infants in the post-algorithm group. We present their characteristics in the Supplemental Digital Content Table, available at http://links.lww.com/PQ9/A150. The cost analysis showed a saving of over $300 per infant not treated with antibiotics in the post-algorithm group (see Supplemental Digital Content at http://links.lww.com/PQ9/A149 for Figure 2).

## DISCUSSION

There is a lack of precision and consistency in the clinical diagnosis of chorioamnionitis. The signs and symptoms are nonspecific and do not necessarily indicate intrauterine infection.^4^ The categorical risk factor assessment recommended by COFN and CDC with empiric antibiotic treatment for all the infants exposed to chorioamnionitis will result in a large number of uninfected infants being treated based on the prevalence of chorioamnionitis.^15^ The consequence of this management guideline may include mother-infant separation for a variable period. This separation may adversely affect the normal metabolic adaptation of the infant, and disturb breastfeeding and skin-to-skin care.^16,17^ Antibiotic exposure in infancy may alter the gut microbiome and immune programming.^18^ Studies have demonstrated an association between early antibiotic exposure and increased risk of later childhood health problems, such as asthma, allergies, obesity, and autoimmune disorders.^19^ Therefore, it is important to find safe and effective ways to identify the risk of EOS and limit antibiotic exposure in infants exposed to maternal chorioamnionitis.^20,21^

To assess the infants who are at increased risk of EOS without unnecessary laboratory utilization and antibiotic administration, 2 main approaches have been described. Multivariate risk assessment, using a combination of maternal risk factors and an infant’s clinical condition, determine the level of risk, which directs the management of the infant.^22,23^ The use of this multivariate model, such as neonatal EOS risk calculator, may reduce the use of laboratory tests and empiric antibiotics in infants exposed to maternal chorioamnionitis.^24,25^ However, there is a risk of missing a few infants with culture-positive EOS or delaying antibiotic use.^26–29^ Moreover, about one-third (23 to 35%) of asymptomatic infants may receive antibiotics unnecessarily.^28,29^ Experts have raised concerns regarding the ability of an EOS risk calculator to safely reduce antibiotic use without missing a case of EOS.^30^ Also, the implementation of the EOS risk calculator requires training, may increase nursing and provider workload and lead to medical errors due to miscalculation of EOS risk.^12^

The second approach is risk assessment, primarily based on an infant’s clinical condition. At-risk infants are clinically monitored in the hospital at least 48 hours after birth with an intervention (laboratory testing and treatment) only if signs of sepsis become apparent. Studies have shown no difference in clinical outcomes with fewer laboratory tests and less antibiotic use.^31,32^ In a more recent quality report, clinical monitoring of infants exposed to chorioamnionitis resulted in decreased laboratory testing and 55% reduction in antibiotic use with no culture-positive EOS cases.^33^ The authors did recognize the limitation that their results are not generalizable to centers where newborn hospitalists are not available around the clock to perform serial clinical exams. Clinical monitoring requires more time and effort by healthcare providers, and if the monitoring occurs in the NICU, it may lead to mother-infant separation and increased healthcare costs. There is a possibility of unnecessary antibiotic usage due to nonspecific clinical signs, which may not reflect infection. Moreover, multiple studies demonstrated that a large number of infants who develop EOS might remain asymptomatic at birth. In a large multicenter study, 13% of infants exposed to maternal chorioamnionitis with EOS remained asymptomatic at 6 hours after birth, including 9% of whom had no symptoms at 72 hours of life.^15^ Another study reported that 50% of infants with culture-positive EOS were asymptomatic at birth.^26^ The initiation of treatment later in the course of sepsis may lead to worse outcomes.

Several studies have shown the lack of sensitivity, specificity, and poor positive predictive value of CBC and CRP in the evaluation of EOS.^34^ Abnormal lab values in asymptomatic infants with culture-negative sepsis may lead to unnecessary NICU admission, prolonged antibiotic exposure, and increased length of stay.^6^ However, both CBC and CRP have an excellent negative predictive value.^8^ Among CBC indices, the I:T has a high negative predictive value for neonatal infections.^35^ If I:T is <0.2 at 6 to 12 hours of life, the likelihood of having an infection is very low. Combining clinical examination with laboratory testing can enhance the sensitivity of detecting EOS in an infant. One of the recently published studies concluded that laboratory measurements along with clinical monitoring in all asymptomatic chorioamnionitis-exposed infants would have decreased antibiotic exposure to as low as 9%–12% and identified all the culture-positive EOS compared with using EOS risk calculator.^29^

After reviewing the available published evidence, we developed an institutional algorithm that combines both laboratory testing and clinical monitoring for the management of asymptomatic neonates exposed to maternal chorioamnionitis. We believed this consistent approach best suits our practice conditions, local resources, and structure. We closely monitored the results of the practice change and significantly decreased infant antibiotic utilization in the MBU by 95% (from 96.8% to 4.5%) without an increase in HLOS or incidence of NICU admissions. None of the infants had culture-positive EOS or readmissions for sepsis within 7 days of discharge. This outcome was possible due to the interprofessional collaboration between newborn providers and the nursing staff who interpreted the laboratory results and clinically monitored the infants every 4 hours during the hospital stay. Moreover, this intervention did not result in the separation of the mother and her infant, and it reduced costs (over $300 per infant not treated with antibiotics).

This QI study has several limitations. We depended on clinical diagnosis of chorioamnionitis by the obstetric provider, as only a few placentas were sent for histological evaluation. As this study is from 2 hospitals from the same hospital system covered by the same newborn provider group, results may not be generalizable. There was a possibility of treating asymptomatic infants depending on the second I:T > 0.2, but only 1 infant in the entire post-algorithm cohort had that abnormality leading to treatment with antibiotics for 48 hours. We recognize the limitations of small sample size and low incidence of culture-positive EOS; however, we felt it was important to share our experience with creating an effective institutional algorithm that decreased antibiotic utilization in the MBU without an increase in cost or adverse effects.

## CONCLUDING SUMMARY

By following a consistent institutional algorithm of monitoring chorioamnionitis-exposed infants with clinical and laboratory evaluation, antibiotic utilization in MBU decreased significantly without an increase in HLOS, NICU admissions, or readmissions for sepsis within 7 days of discharge. This algorithm combining both laboratory testing and clinical monitoring could be optimal to manage these asymptomatic infants with a low incidence of EOS. Larger studies are needed to validate this approach.

## DISCLOSURE

The authors have no financial interest to declare in relation to the content of this article.

## Supplementary Material
