# Supplementary material for: An Institutional Approach to the Management of Asymptomatic Chorioamnionitis-Exposed Infants Born ≥35 Weeks Gestation
Source: Pediatr Qual Saf. 2019 Dec 5;4(6):e238. doi: 10.1097/pq9.0000000000000238 (PMC6946240; doi:10.1097/pq9.0000000000000238)
Supplement: Supplementary file 1 [file pqs-4-e238-s001.pdf]

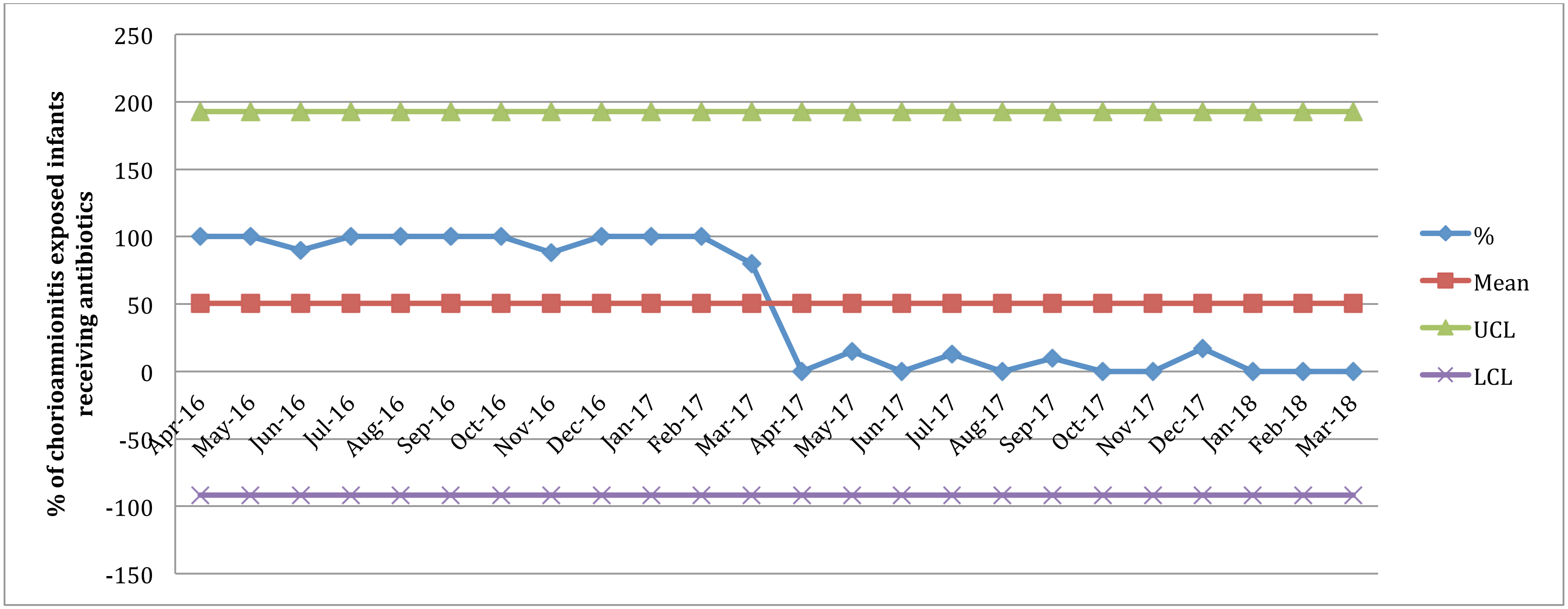

Supplemental Digital Content Figure 1: Statistical Process Control chart pre and post algorithm implementation depicting improvement

Chiruvolu A. An Institutional Approach to the Management of Asymptomatic Chorioamnionitis-Exposed Infants Born ≥35 Weeks Gestation
