# Supplementary material for: An Institutional Approach to the Management of Asymptomatic Chorioamnionitis-Exposed Infants Born ≥35 Weeks Gestation
Source: Pediatr Qual Saf. 2019 Dec 5;4(6):e238. doi: 10.1097/pq9.0000000000000238 (PMC6946240; doi:10.1097/pq9.0000000000000238)
Supplement: Supplementary file 2 [file pqs-4-e238-s002.pdf]

Item	Cost/unit	Pre-algorithm (123)			Post-algorithm (111)			Average cost saving/Infant
		Number of units	Cost	Average cost/Infant	Number of units	Cost	Average cost/Infant	
Antibiotic doses (ampicillin/gentamicin)	$9.32	702	$6,542.64	$53.19	30	$279.60	$2.52	$50.67
Nursing costs for time spent per infant starting peripheral intravenous line (IV) and administering antibiotics: Registered Nurse (RN) - 5 hours ($36.59/hour)	$182.95	119	$21,771.05	$177.00	5	$914.75	$8.24	$168.76
Pharmacist dispensing antibiotics: 50 minutes x $60.78/hour	$50.65	119	$6,027.35	$49.00	5	$253.25	$2.28	$46.72
Pharmacy tech delivering antibiotics: 80 minutes x $19.95/hour	$26.60	119	$3,165.40	$25.73	5	$133.00	$1.20	$24.54
IV supplies	$21.60	119	$2,570.40	$20.90	5	$108.00	$0.97	$19.92
Lab (CBC/CRP)	$10.00	194	$1,940.00	$15.77	135	$1,350.00	$12.16	$3.61
	Total			$341.59			$27.36	$314.23

Supplemental Digital Content Figure 2: Cost analysis. For each chorioamnionitis-exposed infant not treated with antibiotics in the post-algorithm cohort, the cost savings were $314.23

Chiruvolu A. An Institutional Approach to the Management of Asymptomatic Chorioamnionitis-Exposed Infants Born ≥35 Weeks Gestation
