# Supplementary material for: An Institutional Approach to the Management of Asymptomatic Chorioamnionitis-Exposed Infants Born ≥35 Weeks Gestation
Source: Pediatr Qual Saf. 2019 Dec 5;4(6):e238. doi: 10.1097/pq9.0000000000000238 (PMC6946240; doi:10.1097/pq9.0000000000000238)
Supplement: Supplementary file 3 [file pqs-4-e238-s003.pdf]

Supplemental Digital Content Table: Characteristics of NICU admissions

Chiruvolu A. An Institutional Approach to the Management of Asymptomatic Chorioamnionitis-Exposed Infants Born ≥35 Weeks Gestation

Pre-algorithm: 3 NICU admissions

Reason	CBC/CRP	Blood culture	Antibiotic duration (hours)	Length of stay (days)
Tachypnea	Normal	Negative	48	2
Hypoglycemia	Normal	Negative	48	4
Hypoglycemia	Normal	Negative	48	4

Post-algorithm: 4 NICU admissions

Reason	CBC/CRP	Blood culture	Antibiotic duration (hours)	Length of stay (days)
Hypoglycemia	Normal	Negative	48	4
Tachypnea	Abnormal	Negative	48	3
Tachycardia	Abnormal	Negative	48	5
Tachypnea	Normal	Negative	48	2
